# Primary care for diabetes mellitus patients from the perspective of the care model for chronic conditions^1^

**DOI:** 10.1590/1518-8345.1474.2882

**Published:** 2017-03-09

**Authors:** Maria Aparecida Salci, Betina Hörner Schlindwein Meirelles, Denise Maria Guerreiro Vieira da Silva

**Affiliations:** 2 PhD, Adjunct Professor, Departamento de Enfermagem, Universidade Estadual de Maringá, Maringá, PR, Brazil.; 3 PhD, Associate Professor, Departamento de Enfermagem, Universidade Federal de Santa Catarina, Florianópolis, SC, Brazil.; 4 PhD, Full Professor, Departamento de Enfermagem, Universidade Federal de Santa Catarina, Florianópolis, SC, Brazil.

**Keywords:** Diabetes Mellitus, Primary Health Care, Health Services Evaluation

## Abstract

**Objective:**

to assess the health care Primary Health Care professionals provide to diabetes mellitus patients from the perspective of the Modelo de Atenção às Condições Crônicas.

**Method:**

qualitative study, using the theoretical framework of Complex Thinking and the Modelo de Atenção às Condições Crônicas and the methodological framework of assessment research. To collect the data, 38 interviews were held with health professionals and managers; observation of the activities by the health teams; and analysis of 25 files of people who received this care. The data analysis was supported by the software ATLAS.ti, using the directed content analysis technique.

**Results:**

at the micro level, care was distant from the integrality of the actions needed to assist people with chronic conditions and was centered on the biomedical model. At the meso level, there was disarticulation among the professionals of the Family Health Strategy, between them and the users, family and community. At the macro level, there was a lack of guiding strategies to implement public policies for diabetes in care practice.

**Conclusion:**

the implementation of the Modelo de Atenção às Condições Crônicas represents a great challenge, mainly needing professionals and managers who are prepared to work with chronic conditions are who are open to break with the traditional model.

## Introduction

Diabetes mellitus is the group of chronic conditions responsible for the main causes of death around the world and is considered one of the greatest health problems^1^. In that sense, Brazilian and international studies have discussed actions that can help to control the advance of this disease and its complications, and that often derive from care that does not consider the particularities of chronicity, with inadequate care models that are focused on curing the disease, present in the logic of the biomedical model^2^ ^-^ ^4^.

Starting from an important international discussion at the end of the 20^th^ century and the start of the 21^st^ century about health care models that take into account the needs of people with chronic conditions, new care models have been created, such as the Chronic Care Model^5^, developed in the United States, which has been widely used in different countries around the world, with reports of successful experiences^6^.

The *Chronic Care Model* was the main reference for the construction of the *Modelo de Atenção* às *Condições Crônicas* (MACC)^7^, elaborated for the Brazilian reality and health context. It considers the particularities of chronicity and of the living conditions resulting from illness, the contexts related and inter-related in this process and the person, his family, such networks, health care networks, services, professionals, management and policies^7^.

The Brazilian Health Department has used this model as a reference for the establishment of care policies for people with chronic conditions, such as the development of the Health Care Networks (HCN). Although the assessments of the implementation of this model are limited, as few studies have been developed thus far, research has revealed that it is fit in care delivery to chronic patients^3^ ^,^ ^8^. Specifically concerning health care for diabetes patients, we found no studies that permitted assessing whether this model has served as a reference for health professionals and what changes it has promoted by offering a specific structure for health care provision to chronic patients. In that context, the objective in this study was to assess the health care Primary Health Care (PHC) professionals provide to diabetes mellitus patients from the perspective of the MACC. 

## Method 

Qualitative study, using Complex Thinking and the MACC as a theoretical framework and assessment research as a methodological framework. Thirty-eight health professionals participated in the study, who are engaged in health care for diabetes patients in PHC: 29 members of five Family Health Teams (FHT) - five physicians, five nurses, four auxiliary nurses and 15 Community Health Agents (CHA); six members of the Family Health Support Center (FHSC) - two pharmacists, one physical educator, one psychologist, one social worker and one nutritionist; and three managers - two from the Primary Health Care Service (PHCS) and one linked to the city.

The selection was based on theoretical sampling. First, data were obtained from the Primary Care Information System on the FHT with the largest number of diabetes patients registered in their coverage area. The FHSC professionals and managers were indicated as reference persons for the health professionals on these FHT. 

First, the directors of the PHCS where the FHT worked were contacted to presented the researcher and get acquainted personally with possible participants, to which the study objectives and motives were explained.

The data collection techniques included: interview, observation and consultation of histories. For each, scripts were elaborated in line with the research objective.

Intensive individual interviews were held with each participants, which took between 30 and 150 min and were recorded and fully transcribed. The participants were between 25 and 59 years old and had worked in PHC between four months and 14 years. The observation involved moderate participation and took place during 18 group sessions for diabetes patients developed by the FHT professionals, corresponding to 40 hours for that activity, which were registered in a field diary. Twenty-five histories were consulted, selected by the nurses, who indicated five histories of diabetes patients monitored by their team. The data collection took place during six months (between December 2013 and May 2014) and was terminated in compliance with information saturation criteria. 

To analyze the data, the combination of the adopted techniques permitted triangulation in the analysis process, which was guided by directed content analysis. The MACC served as a reference, in view of its range (micro, meso and macro)^7^, and the protocol proposed in the Health Department policy for diabetes: Primary Care Notebooks - Strategies for care to chronic patients - Diabetes Mellitus^9^.

ATLAS.ti software version 7.1.7 was used, license 58118222, as a technological tool to support the organization and coding of the interview. The analysis of these data involved coding, the initial analysis when the data were opened and the codes originated in the interviews were identified; and the reorganization of the data around axes of interest, guided by the theoretical frameworks adopted. The data on the observations and histories were analyzed similarly, but were used to support or clarify the participants' statements about the care they provided. To present the interview results, an identification was used, indicating the profession and/or function, followed by the letter P and a number, corresponding to the inclusion of the interview in ATLAS.ti.

All participants signed two copies of the Free and Informed Consent Form. Authorization was obtained from the Permanent Ethics Committee for Research Involving Human Beings at *Universidade Federal de Santa Catarina* and the City Health Department authorized the research. 

## Results

**Figure 1:**
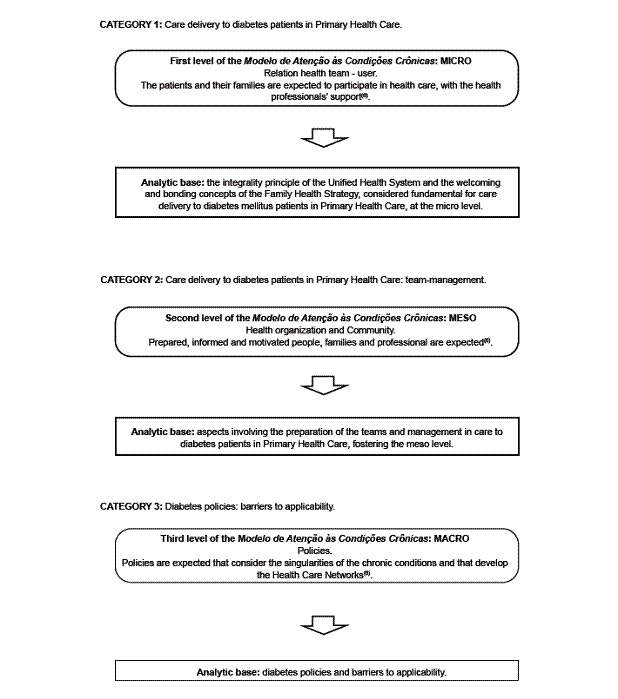
Categories and their relations with the MACC levels and the analytic bases

The study results are presented in three categories (Figure 1), each related to one of the levels of the MACC and one analytic base: *Care delivery to diabetes patients in PHC,* corresponding to the micro level; *Care delivery to diabetes patients in PHC: team-management,* corresponding to the meso level; and, *Policies for diabetes: barriers to applicability,* corresponding to the macro level. 

### Care to diabetes patients in Primary Health Care

The large majority of diabetes care was offered collectively, aiming to promote care access to a larger number of users, who were registered in the Registration and Monitoring System of Hypertensive and Diabetic Patients. This was called group care and was organized through quarterly pre-scheduling, favoring this access to the health service and optimizing the professionals' agenda for care delivery to more people with the same disease on predefined dates and times. The dynamics established to hold the groups was care centered on the medical appointment, prescriptive, in which the main activity was to deliver prescriptions and, sometimes, but not systematically, laboratory tests were requested. This moment was marked by a very short period to attend to people, entailing important activity constraints, such as the physical examination.

*The physical examination is done in case of complaint, there is no routine [...]. On group days, I have five minutes to attend to the patient, between explaining all tests and advising on the medication and doing the physical examination... It's impossible to do the physical examination...* (Physician-P30)*.*

There was no planning of the professionals' activities that considered the set of the diabetes patients' needs. This situation entailed implications for the establishment of welcoming and bonding, leading to the lack of early detection of the disease and its complications and the inexistence of systematic monitoring of diabetes patients. The framework for the monitoring was often the presence of severe and irreversible complications.

*My patient arrives, I don't even know if he's diabetic, if he's hypertensive... He comes for medical care when it suits him or when something happens [...] a foot sore, when something happens, a casualty, some doubt, he decompensated... But there is no specific planning yet for these diabetics* (Nurse-P4)*.*

In this group care, people did not get the opportunity to discuss other health problems. They should only restrict themselves to their clinical problems related to the diabetes and hypertension. When mentioning a different complaint, the patient was advised to schedule another appointment to try and solve his problems, entailing an additional demand for the service and difficulties for these people.

The focus in the care the FHT members provided was the physician, without expressing inter or multidisciplinary work. The nurses did not engage in care and systematic monitoring to prevent and reduce chronic complications of the diabetes. Educational activities were offered partially or not at all.

*[...] How will I tell a 70-year-old patient that he needs to clean the vial, aspirate, take the syringe if he no longer has manual dexterity, the skill to puncture there. You explain it, he looks at you and you know he didn't get it. Then you look and think: should I ask if he understood or shouldn't I? Because, if he didn't understand, what if I have to explain everything again...* (Nurse-P4)*.*

Another aspect found was the lack of integration between the professionals and the relatives of diabetes patients, as well as with their social context. It is highlighted that, except for the CHA, no other professional appointed the existence of this engagement and direction in their actions and practices in the care and assistance process for diabetes patients in PHC.

### Care to diabetes patients in Primary Health Care: team-management

The professionals perceived motivation, considered as a requisite for self-care, as something distant from the reality of the current care practices for diabetes patients in PHC. In the observation of the group meetings, the diabetic patients remained seated for a long time, passively, awaiting the physician to call them. Overall, there was no systematic interaction with the professionals and even among them.

*The group seems like a slaughterhouse. They are all sitting, nursing and the CHA do what they need to and then they wait alone for the physician to call them* (CHA-P13)*.*

Another important issue is that not all professionals, particularly at the FHSC and technical professionals, possessed clinical knowledge about diabetes, especially its complications. That caused difficulties for these professionals to act, specifically in educational activities to prevent diabetes-related complications. The CHA created self-training strategies, attempting to read the folders and pamphlets distributed by the Municipal Secretary and Health Department, and also indicated that, when they felt the need, they asked questions to the professionals they considered more knowledgeable.

*I know that the patient can lose his foot and that it can affect his vision, I don't know if there are further complications [...]. I think we should know more about diabetes to advise people correctly. We get no training, what I know is because I ask one, another, there's always a colleague who knows more and I also try to read everything the Secretary sends us* (CHA-P22)*.*

As for the management aspects, the municipal management's difficulty to monitor the care the FHT offer to diabetes patients is highlighted. The local PHCS management could fill this gap who, as part of their function, should supervise the care developed in the places they are responsible for. Nevertheless, the local managers came with party orientations upon the determination of the current public management. Many of them were commissioned agents and assumed their function without education and background experience in health, with great difficulty to engage in more specific aspects, and even to comply with the public policies.

*When I was invited to come to this Service, I got anxious and worried, because what did I know and understand about health? Nothing, nothing... After I came here, I noticed that it worked because there's the organization, structuring, employees... that part was disorganized and I was able to work [...]. I face many difficulties until today, I still ask a lot of help from the nurses, I still need to learn a lot, a lot...* (Local manager/PHCS-P33)*.*

Another aspect to be considered in this care was the lack of actions that identified the mobilization of social resources, with community orientation, for this cause, as proposed in the MACC.

### Diabetes policies: barriers to applicability

As barriers found for the implementation of public diabetes policies, in the care the PHC professionals and managers currently develop, it is highlighted that none of the interviewees that they develop their clinical practices according to the recommendations in the manuals and protocols of the Health Department: *Like, saying that we apply what is in the manual... No!* (Nurse-P2). Particular attitudes marked the conduct adopted, according to the education and experience of each professional, gained based on his relations and interactions with the historical praxes, without any recycling and monitoring of political changes.

*My conducts are based on the baggage that was learned in college and practice over the years in the Family Health Strategy, because theory is different from practice, there is an entire social, family context and everything else, you can't just consider what you see in books* (Physician-P31).

The implementation of the Health Department or the State Secretaries' proposals (protocols, manuals) was a decision of each health professional, without spaces for discussion that would lead to a collective decision of the FHT or the PHCS. Knowledge on the content of the material was at the discretion of each professionals and his personal initiative to read and implement it.

*But why don't you? Because there was no time to read the manual, because I'm alone to do thousands of things. The nurse chooses whether to read and try and change something, but if he doesn't want to know, he continues like that. The protocol, I took it home to read the one about cervical cancer, breastfeeding and I will take the one for hypertensive and diabetic patients. Because, like, the Department issues the protocol, the book arrives her and that's it* (Nurse-P1).

Concerning the HCN, these were not considered a reality of the municipal health system yet, especially due to their weaknesses in the connections among the different points of the network. The health professionals had no specialized/secondary service for reference and support, with great difficulty to forward the patients as needed to adequate care. In addition, when the specialized consultation happened, the counter-referral was still precarious. The user should bring the prescription from the specialist for the PHCS/FHT professional to continue prescribing the proposed drugs; expressing the lack of articulation among the different network points.

## Discussion

According to the study results, group care for diabetes patients was marked by activities that were more focused on attending to the demand for medical appointments. This care was not guided by the health policy, indicating only partial compliance with the care policy for diabetes patients^9^. The actions were developed based on the biomedical model, with great distancing from the necessary practices for chronic patients, in accordance with the MACC^7^. Similar situations were also found in other PHC studies, showing that this situation is repeated in different places across the Brazilian reality^10^ ^-^ ^11^. 

Listening, as an element of welcoming, fundamental to establish bonding and considered one of the pillars of therapeutic action, being fundamental for care integrality^12^, was not evident in this care. Individualized monitoring is recommended for diabetes patients, considering that the context of each patient and the way (s)he lives with the disease are essential for care that is intended to maintain the glucose levels under control and promote patients' quality of life^9^ ^,^ ^13^. 

Orientation, as a health education instrument, is fundamental to allow people to practice self-care^9^ ^,^ ^14^ ^-^ ^15^, in order to have a healthy and productive life. Studies that analyzed the health practice and health education concepts highlight the need to further value the role of health professionals as drivers of change in health education and in the health model they are inserted in^15^ ^-^ ^16^.

The lack of integration between the professionals and the diabetes patients' family, as well as with their social context, indicates the lack of convergence between health care practice and the principles of the Family Health Strategy (FHS), integrality and the MACC, in which the family should be involved in the users' care plan^7^.

The shortcomings the CHA expressed concerning the limited knowledge about diabetes are in line with another study that also appointed the need to better equip this professional category, which plays a fundamental role in the communication and relationships with the users^17^, even when considering that the CHA execute no clinical activities.

In the performance of its functions, the local management faces difficulties to understand and act in view of the complexity of a health services. Traces were found of reductionist, isolated and simplified activities in the management and political process, such as the non-mobilization of social resources with community orientations towards this cause, as proposed in the MACC^7^.

The managers' activities demonstrate a lack of articulation with the health professionals and the PHC users' reality. They need adequate training, appropriating themselves of the knowledge on health actions and their countless interrelations with other social sectors, and even within the health system. The fragmented perspective on the reality prevents them from apprehending its multiple facets^(18)^. Another study developed in PHC also identified management problems, highlighting a strong party-political influence with patronage characteristics and a lack of qualified professionals^10^.

The health professionals do not use the Health Department documents as a reference for their actions for care to diabetes patients. That is so because the implementation of a health policy meets with countless bottlenecks at the local level, permeated by strategic aspects, interests and multiple actors, considered as determining aspects for the success or failure of this policy^19^.

In view of these connotations and the organizational systemic complexity, multiple weaknesses were identified in care delivery to diabetes patients in PHC, especially when analyzed from the perspective of the MACC. According to the complexity paradigm, different situations came up that point to a fragmented, reductionist, contradictory organization that is disjunctive of the underlying policies, in which different established disorders can be identified. In view of the basic principles of complex thinking, however, which is present in all living phenomena, any disorder can produce a new order and establish a new organization^18^.

Brazilian studies that picture the applicability of the MACC evidence that, after its implementation, care for the people improved, with enhanced treatment compliance by the patients^3^ and the introduction of new care strategies by the health professionals, including actions for self-care, motivational interview and operative group^8^. This reality converges with international experiences, in countries that adopted a new specific care model for chronic conditions, which affirm their efficacy and efficiency to assist this population in its singular and plural aspects, expressed in their health-disease processes^6^ ^,^ ^20^.

As limitations, we register that this study only considered one city and assessed the model focusing on diabetes as a chronic illness. Nevertheless, the professionals do not acknowledge the MACC as an orientation. 

For the MACC to be a reality in the primary health care network and be effective for diabetes care, changes at different levels of the care model are fundamental, including more specific preparation, besides changes in the health care structure, influencing the way health professionals and managers act. 

## Conclusion

Based on the assessment of the PHC professionals' health care for diabetes patients from the perspective of the MACC, it could be concluded that the implementation of this model is a great challenge, marked by the need for professionals and managers who are prepared to work with chronic illnesses and who are open to break with the traditional model; and policies that grant conditions to operate this model at the micro, meso and macro levels. 

These results can contribute to the reorganization of care for diabetes patients in PHC, with notes for the health professionals, especially the nurses, who should focus on actions to recover the paradigms, concepts and objectives of the care models they are inserted in (PHC and FHS), which should underlie their practices. For the managers, they appoint the need to consider measures that can grant support for the professionals to develop integral care. For the public diabetes policies, they signal the need to propose control and monitoring strategies of the care offered at the base, with measures that guarantee, beyond access, the quality of care for diabetes patients.
